# Operation for United Fracture of the Olecranon by Opening the Joint, and Wiring the Fragments to the Ulna; Bony Union

**Published:** 1880-10

**Authors:** 


Operation for United Fracture of the Olecranon by Opening the Joint, and Wiring the Fragments to the Ulna; Bony Union. (Under the care of Mr. William Rose.)

For the following notes we are indebted to Mr. John Phillips, house-surgeon:

W. M., aged twenty, was admitted into Fisk ward on March 31. Early in January he fell down on his left elbow. Great swelling followed, and he was treated by his medical attendant for a simple sprain. He noticed, however, that as the pain and swelling subsided he could not extend the forearm. In the beginning of March he attended King's College Hospital as an out-patient under Mr. Rose. A fracture of the left olecranon was detected with about half an inch separation between the broken surfaces. The forearm was extended and bandaged upon a splint in order to relax the triceps, so as to bring the olecranon down to position. As this was followed by no marked improvement, Mr. Rose operated on April 1.

A longitudinal incision three inches in length was made over the seat of fracture, and the joint opened. The olecranon was freed with a few touches of the knife from its lateral attachments, and drawn down to the ulna without difficulty. The fibrous tissue which had formed between the broken surfaces was carefully dissected away, and the opposing surfaces of bone were scraped with a chisel until they were quite bare. A hole was then drilled obliquely in the ulna, and another exactly corresponding to it was made in the olecranon, care being taken to bring out the point of the awl, on the rough surfaces just outside of the articular cartilage. A strong silver wire loop was then passed through the holes thus made, and drawn tight. The broken fragments were thereby brought closely and firmly together and the wire was entirely outside the joint. Horsehair drainage was used, and the wound was closed with carbolized silk sutures. Gauze dressings were applied, and the limb was put on a splint with the forearm extended. Strict antiseptic precautions were observed throughout.

The after-progress of the case was highly satisfactory. The joint remained perfectly quiet, the patient complained of no pain, and the temperature never rose above 99.4°, nor his pulse above 84.

The antiseptic dressings were changed only seven times in the five weeks which elapsed before the silver wire was removed. The joint was moved the tenth day, and on the seventeenth day from the operation the patient was able to extend the forearm. After this the action of the triceps steadily improved, and the patient left the hospital on May 13 with evidently firm bony union.

Mr. Rose remarked that he was led to undertake this operation of opening the joint and wiring the fragment, not because the fracture was ununited, but because the arm had lost all power of extension in consequence of the complete laceration and displacements of the lower attachments of the triceps muscle. He would not recommend the operation in every case of fracture of the olecranon. It was not an uncommon occurrence to find a considerable amount of extension in such cases. This was to be explained by assuming that, though the olecranon had been detached, the fibrous insertion of the triceps had not been entirely severed.

---

LITERATURE AND MEDICINE is the title of an article in the July *Eclectic.* It treats of the various points at which the two professions touch. Among others is the kind tendance given to men of genius by those whose care and duty it is "to stand between man and his doom. "Who can forget Dryden's grateful acknowledgement of the services of Hobbes and Guibbons? or Cheselden's goodness to Pope? or Meade's to Gay? or Arbuthnot's to every literary man with whom he came in contact? 'There is no end of my kind treatment from the faculty,' writes Pope, a few weeks before he died; 'they are in general the most amiable companions and the best friends, as well as the most learned men I know.'"

